# Exploring Barriers: How to Overcome Roadblocks Impeding the Provision of Postabortion Care to Young People in Togo

**DOI:** 10.9745/GHSP-D-18-00437

**Published:** 2019-08-22

**Authors:** Stembile Mugore

**Affiliations:** a IntraHealth International Inc., Chapel Hill, NC, USA.

## Abstract

**Background:**

Family planning counseling and provision of a variety of contraceptive methods during postabortion care (PAC) can increase voluntary contraceptive use and prevent unintended pregnancies, helping to reduce maternal morbidity and mortality. Youth in Togo, however, face many barriers to accessing contraceptives during PAC including provider bias, parental consent policies, stigma, and pressure on young people to have children. This article describes the lessons learned from follow-up with providers who were trained on PAC to build their willingness and skills in offering youth-friendly PAC.

**Methods:**

With assistance from the Evidence to Action (E2A) Project, Togo’s Division for Maternal and Infant Health and Family Planning (DSMI/PF) systematically applied IntraHealth International’s Optimizing Performance and Quality (OPQ) approach to improve the quality of PAC at 5 health facilities from 2014 to 2016. OPQ identified gaps and helped establish solutions to shortcomings that hinder youth’s access to contraceptive choice during PAC. To address these shortcomings, staff trained providers to improve their knowledge, attitudes, and skills with regard to (1) medical eligibility criteria for contraceptive use to remove age as justification for denying contraceptives; (2) AFPAC to increase the ability to consider gender-based violence and to encourage client-focused and age-sensitive counseling; and (3) monitoring through disaggregation of data by age, method, and parity for data-driven planning and delivery of AFPAC.

**Results:**

Providers reported improved attitudes and empathy toward young PAC clients, efforts to screen for exposure to sexual and gender-based violence, and willingness to explore reproductive health needs and assist clients in choosing contraceptive methods. Previously, no youth PAC clients chose contraceptives during PAC or returned to the facility for routine follow-up. After providers received OPQ training, they treated 775 PAC clients in 5 health facilities between June and December 2016; 351 were ages 14–24, of whom 142 (40.5%) chose a modern contraceptive. The most common method selected by youth was oral contraceptive pills (44%), followed by implants (41%) and injectables (15%). Of the 142 youth PAC clients who chose a method, 86 (58.5%) returned for the 7-day follow-up.

**Conclusions:**

Training providers to offer AFPAC along with the application of OPQ to improve the quality of PAC increases voluntary contraceptive uptake, including implants, among youth who make up a substantial portion of all PAC clients. Incorporating AFPAC in family planning/reproductive health policies, norms, protocols, and PAC training, as Togo’s DSMI/PF did, is an important element to ensuring institutionalization of the practice.

## Abstract (precis)

Before providers were trained in offering youth-friendly postabortion care (PAC), including provision of voluntary contraceptive methods, no youth PAC client chose a modern method before leaving the facility. After training, over a 6-month period 41% of youth PAC clients chose a modern method, most commonly oral contraceptive pills followed by implants and injectables.

## BACKGROUND

Adolescent girls—15–19 years old—are twice as likely to die during pregnancy or childbirth as women 20 years of age or older. Further, girls younger than 15 are 5 times more likely to die during pregnancy or childbirth than women 20 years of age or older. Of the approximately 252 million adolescent girls in developing regions, about 38 million are sexually active and the majority of these girls (23 million) have an unmet need for family planning. Approximately half of adolescent pregnancies are unintended,^1^ and the World Health Organization (WHO) estimates that 2–4.4 million adolescents in developing countries undergo unsafe abortions performed by unskilled providers under dangerous and unhygienic conditions each year.^2^

Adolescent girls have a high unmet need for contraception for multiple reasons, including their own misperceptions that they are at low risk of pregnancy due to infrequent sex and being unmarried, fear of side effects, and social stigma around being sexually active. Studies that have reviewed reasons for adolescent girls encountering barriers to obtaining contraception include providers’ judgmental attitudes and bias, a lack of privacy and confidentiality, and a lack of sound policies and guidelines on postabortion care (PAC) and family planning services.^3^

Many countries lack guidelines regarding the provision of PAC, including high-quality voluntary family planning services, to adolescents although the WHO recommends provision of quality PAC to adolescents as an international standard. Quality PAC is defined as including emergency treatment for abortion complications; access to voluntary family planning services that offer counseling for informed choice and a wide range of contraceptive methods inclusive of long-acting reversible contraceptives (LARCs); referrals for other reproductive health services, such as treatment for sexually transmitted infections and screening for cervical cancer, regardless of whether a pregnancy ended with miscarriage or an abortion; and community empowerment through community awareness and mobilization.^4^ High maternal morbidity and mortality highlight the need for voluntary family planning services and yet adolescents often do not use these services, underscoring the importance of including adolescent friendly services with quality PAC. Interventions that increase voluntary modern contraceptive use and reduce the unmet need for contraception among adolescents would break the cycle of repeated unintended pregnancies, reducing them by 6 million every year. Achieving this goal would avert 3.2 million abortions and 5,600 maternal deaths globally each year.^1^

Adolescent and youth reproductive health (AYRH) indicators in Togo reveal a great need for quality PAC and other evidence-based interventions for encouraging voluntary contraceptive use to reduce unintended pregnancies among adolescent girls. Togolese youth who are 25 years or younger compose the majority (61%) of the total 6.2 million population. Among Togolese women and girls, the unmet need for contraception is highest among adolescents, with 42% of sexually active 15- to 19-year-olds lacking access to contraception compared with 40% of sexually active 20- to 24-year-olds. Togolese girls become sexually active early: 11% before age 15, 47% before age 18, and 71% by age 20. Among all women of reproductive age, modern contraceptive use is extremely limited. The prevalence of modern contraceptive use is 17% among married or in-union women 15–49 years and 12% among unmarried, sexually active adolescents.^5^ Among the married women 15–49 years old who use contraception, many choose injectables (7%) and implants (5%). In addition, according to a 2010 study by the Ministry for the Advancement of Women, 5.5% of girls ages 9–18 are victims of sexual violence. More than 40% of adolescent girls and young women have suffered some type of physical violence in their home, while more than 89% have experienced psychological abuse at home.

Since 2014, the Evidence to Action (E2A), supported by the U.S. Agency for International Development and under the leadership of Togo’s Division for Maternal and Infant Health and Family Planning (DSMI/PF), has worked to increase voluntary access to family planning services during PAC, particularly among adolescents. From 2014 to 2016, E2A applied IntraHealth International’s Optimizing Performance and Quality (OPQ) approach to improve the quality of PAC at 5 high-volume health facilities—2 regional hospitals, 2 district hospitals, and 1 health center. Efforts included provider trainings meant to:

- Update the contraceptive technology available, remove barriers that prevent clients from accessing contraceptives, and broaden the range of contraceptives offered (including LARCs)
- Support counseling for voluntary informed contraceptive choice regardless of age, marital status, or whether the abortion was induced or spontaneous
- Improve recordkeeping and use of data to monitor quality of PAC being provided

The OPQ approach required managers of maternity and family planning units to provide supportive supervision and to help identify gaps and weaknesses in PAC service delivery and ways to address these gaps. The managers were engaged in developing solutions to problems and facilitating peer meetings to review progress and lessons learned, reinforce skills, and address common performance gaps.^6^

## METHODS

First, a team comprising DSMI/PF and E2A conducted a needs assessment to identify current practices and barriers to provision of PAC, including counseling, family planning, and availability of a broad range of contraceptive methods to foster voluntary informed choice. The team E2A reviewed PAC registers at each of the 5 facilities for the period from July 2014 to February 2016 to assess the number of adolescents and youth ages 15–24 years seeking PAC. Individuals were grouped according to age and parity, and family planning policies and PAC guidelines with regard to the management of adolescent PAC clients were reviewed.

Interviews were conducted with facility providers to establish practices related to provision of PAC for adolescent and youth clients, and the facility was observed to assess the organization of services for youth friendliness and the structural barriers that hinder youth from accessing voluntary family planning services.

Following the needs assessment, a set of interventions, described below, were implemented to address the identified barriers, including institutionalization to facilitate scale-up. Also, during the training a written questionnaire was administered pre- and post-training to assess the impact of the training.

### Adaptation of a Global Training Module

In 2012, the PAC Consortium developed a Youth-Friendly PAC Supplemental Training Module^7^ for comprehensive PAC training. The module, intended to improve providers’ abilities to offer high-quality PAC to adolescents, includes the following sessions:

- Overview of youth-friendly PAC supplemental training module
- Overview of adolescence
- Youth-friendly PAC
- Counseling of adolescent PAC clients
- PAC procedures for adolescent clients
- PAC contraception for adolescent clients
- Referral for other reproductive health services

Between January and May 2016, E2A worked with EngenderHealth, the EngenderHealth-led Agir pour la Planification Familiale (Agir/PF), and the DSMI/FP to adapt the training module developed by the PAC Consortium for use in Togo and translated it into French. The resultant AFPAC Training Module for Togo was then updated using WHO’s 2015 *Medical Eligibility Criteria for Contraceptive Use*^8^ and resources from the Postabortion Care website.^9^ It also incorporated statistics and youth-related reproductive health guidelines from Togo.^10^ A new session, “Undertanding and detecting sexual and gender-based violence among teenage PAC clients,” was developed to address judgmental attitudes among providers, such as the belief that all young unmarried PAC clients are promiscuous. The session was intended to create awareness among providers about sexual and gender-based violence (SGBV), increase sensitivity about it (particularly with regard to young PAC clients), and encourage SGBV counseling and support.

### Provider Training

In May 2016, trainers from DSMI/PF and Association Togolaise pour le Bien Etre Familiale (ATBEF) were oriented on the AFPAC Training Module and prepared for their role in cofacilitating the 3-day training workshop. Doctors, clinical officers, and midwives constituted the 27 training participants from the 5 E2A-supported facilities as well as facilities in 3 regions supported by Agir/PF, ATBEF, DSMI/PF, and the AYRH division of the Ministry of Health. The training focused on improving participants’ attitudes toward adolescent PAC clients; integrating gender considerations into the provision of services; and developing skills to support voluntary, adolescent- and youth-friendly family planning counseling and provision of a wide range of contraceptive methods, including LARCs. This training marked the first time that the AFPAC Training Module was used in a Francophone country. The module underwent modifications during training, and it was then finalized and handed over to the DSMI/PF. A written questionnaire was administered pre- and post-training to assess improvements in knowledge after the training (N=27).

### Reorganization of Services

Immediately after the training, E2A and DSMI/PF continued to provide onsite and remote support to the 5 health facilities to help reorganize services to ensure confidentiality in the provision of youth-friendly services. Efforts were also made to help providers identify and establish linkages with other facilities in their districts that offer AYRH and SGBV services.

### Data Collection, Analysis, and Use

Between June and December 2016, monthly data from PAC registries were entered into a Microsoft Excel database to track progress in PAC counseling and method provision. Data were disaggregated by age, parity, type of abortion, whether the client was counseled, the type of contraceptive method she selected, and whether she returned for the routine 7-day follow-up visit. E2A analyzed and used these data to monitor changes in counseling, method uptake, and return for follow-up among adolescent clients. The 5 facilities received remote support for data use and quality improvement.

### Preparing for Scale-Up

DSMI/PF expressed interest in both vertical (government ownership) and horizontal (geographic expansion) scale-up to improve contraceptive method choice among PAC clients. DSMI/PF therefore prioritized updates to policies, norms, and protocols to address the lack of clear guidance regarding provision of PAC. The lack of such guidance had resulted in provider-driven policies based on misperceptions about AYRH, and these policies contributed to the barriers encountered by adolescents and youth when trying to access PAC. Agir/PF was included in adaptation of the training module and cofacilitation of the workshop because project leadership expressed interest in scaling up of AFPAC in Togo and possibly expanding the AFPAC training to other West African countries.

In 2017, E2A, EngenderHealth, Agir/PF, and DMSI/FP updated Togo’s national family planning policies, norms, and protocols to align with the AFPAC module and to support systematic scale-up. Providers from the 5 facilities participated as a resource in updating the guidelines to share practical experiences and make recommendations about key elements that should be included. Examples include parental consent for treatment of abortion complications (particularly when the adolescent client is very sick and needs referral or surgery), guidelines on SGBV, and guidelines on the use of OPQ for quality improvement. DSMI/PF and Agir/PF disseminated the updated policies, norms, and protocols to regional directorates in Togo along with technical and policy updates on management of adolescent and youth PAC clients and MEC for contraceptive use.

## RESULTS

### Pre-Intervention PAC Service Delivery Data

The review of the monthly PAC register entries from July 2014 to February 2016 showed that young clients (15–24 years of age) who were unmarried and pregnant for the first time tended to be the only clients recorded as having complications related to unsafe abortion. None of these clients were counseled, obtained contraception, or returned for routine follow-up at any of the 5 facilities. Regardless of age, married clients who had previous pregnancies were recorded as having had complications related to spontaneous miscarriage; these clients were counseled and could select a contraceptive method.

### Pre-Intervention Provider Knowledge, Attitudes, and Competencies

Providers at the 5 health facilities had already received technical support on the use of OPQ to improve quality PAC to expand method choice. They had also received a contraceptive technology update and were sensitized on the importance of removing age alone as a medical barrier to contraceptives, including LARCs. The providers were highly competent in the provision of PAC, including the provision of a wide range of contraceptive methods. However, none of the 5 facilities were offering AYRH services, and none of the providers had received AYRH training. They also lacked training on providing SGBV services in the context of PAC. For example, history taking did not include questions on coerced sex and SGBV.^11^

These findings highlighted a need to further explore provider competencies in provision of AYRH services, particularly focusing on their attitudes toward unmarried adolescent clients and structural barriers, such as the high cost of services and compromised confidentiality. The barriers described in the following sections were found to inhibit the provision of quality PAC to adolescents.

#### Provider Attitudes and Bias

Discussions with providers revealed that their bias and judgmental attitudes and the provision of poor-quality services blocked adolescent clients from accessing needed PAC. The providers also cited a policy that parental consent was required for provision of voluntary family planning services to adolescents, although no such policy existed. These providers received a contraceptive technology update and values clarification training, and their service-delivery managers were trained on OPQ. Provider comments prior to training included the following examples:

Young clients are always in a hurry to get services and go home before they are found out. We make them wait, and when we treat them we do not give pain medication. This will make them not come again. They are happy to get rid of the pregnancy and do not show remorse. —Midwife

If they are not accompanied by a parent or an adult, we cannot treat them because we fear getting into trouble should anything happen. We send them away to bring a parent. —Midwife

Giving them a contraceptive method only encourages them to continue to have sex. They have to realize they might be infertile in the future. —Midwife

#### Institutional Barriers

**Policies and guidelines:** Policies and guidelines on managing adolescent clients and the provision of contraception were largely not available at the facilities. When policies and guidelines did exist, they were not explicit about the age of clients that can receive PAC with or without parental consent.

**Cost of services:** Among structural barriers, cost was cited at all the health facilities as a major barrier to adolescents accessing services. The cost for PAC treatment ranged from US$18 to $20. If clients opted to obtain contraception, they had to pay an additional fee: $2 to $3 if they chose an injectable contraceptive, or $7 to $10 if they chose implants. The following comment was illustrative of the cost barrier:

Young clients do not always have money even to pay for the treatment of abortion complications and would therefore not afford to pay for contraceptive methods. —Midwife

**Privacy and confidentiality/organization of services:** Weak infrastructure and poor organization of services compromised privacy, which is a key quality-of-care element in providing services to adolescents. Client reception and the waiting area were typically in the maternity ward, where management of labor and delivery were a priority. Management of PAC clients was only made a priority if they were in shock.

#### Client Management

Manual vacuum aspiration (MVA) is a standard method of treatment in PAC. However, most of the facilities lacked both the necessary equipment and providers trained in the method. Providers typically resorted to manual removal of retained products of conception, which is not recommended because it has a high risk of incomplete removal and is more uncomfortable for the client. Clients were not always offered pain medication.

These findings are consistent with those from a baseline assessment conducted at 48 health centers in Lomé, Sokodé, and Kara by the Agir/PF Project between March and June 2014. That assessment found that most providers:

- Do not believe adolescents should be sexually active, do not offer them contraceptives, and simply encourage them to stop having sex
- Believe that only men and women who have had a child should access contraceptives
- Ask about marital status due to the belief that only married clients should have access to contraceptives and the belief that single adolescent clients should have parental consent to leave the PAC service with a contraceptive
- Believe young married clients should seek spousal consent for contraceptive use despite this action not being a policy directive

### Post-Interventions Results

In January 2017, 7 months after the training, interviews were conducted with some of the AFPAC-trained providers who attended the workshops. The interviews covered reproductive health policies, norms, and protocols to find out how the providers had applied their learning and whether they observed significant changes in provision of services to adolescents. We also used the service delivery registers to capture the type of contraceptive method that clients selected and whether they returned for the 7-day routine follow-up visit. A formal evaluation was not conducted due to budget constraints.

#### Pre-Post Training Knowledge Assessment

**FIGURE 1:**
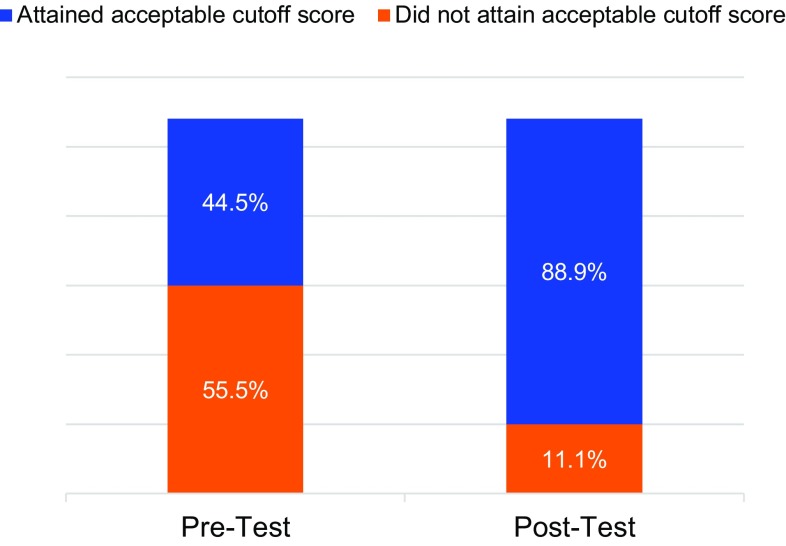
Percentage of Training Participants Attaining Acceptable Cutoff Knowledge Score of 75%, Pre- and Post-Test Results (N=27)

Workshop participants completed a written questionnaire before and after the training. The initial set of results showed that most participants had limited knowledge on how to manage adolescent and youth clients seeking PAC; 55.5% were unable to attain the acceptable cutoff score of 75%. The post-training results revealed a marked improvement in knowledge, with only 11.1% of participants not reaching the 75% cutoff score (Figure 1).

#### Provider Feedback

The trained providers agreed that the workshop had prepared them to manage adolescent clients. They also indicated that the sessions on SGBV were particularly enlightening because they had previously never considered the link between SBGV, age of consent, and PAC among adolescent clients.

The providers reported changed attitudes and increased empathy toward adolescent clients, mentioning that they were no longer judgmental, had greater patience with their young clients, and took time to establish rapport with them. They also mentioned that they were screening for SGBV and providing counseling. However, referral facilities for victims of sexual violence are in major urban areas and therefore not easily accessible for rural clients. Providers shared what they had learned about the importance of AFPAC with peers to foster youth friendliness in the provision of PAC and voluntary family planning services. Some mentioned that they were also applying what they learned from the AFPAC training to provision of maternal health services to adolescent and youth clients. The following provider statements illustrate how services for young clients changed:

[Previously,] providers were in a rush and rude toward youth/adolescents, and young clients did not opt for family planning methods easily and did not come to seek for help. Now there is more use of family planning by postabortion care clients including youth/adolescents. —Midwife

They trust us because we assure them of confidentiality and show them respect and sympathy. We tell them they do not need to bring a parent to leave the PAC service with contraception. —Midwife

At two of the facilities, providers had reorganized the client flow and available space to create separate waiting areas for adolescent and youth PAC clients and to reduce their waiting time.

#### Family Planning Uptake Among Youth PAC Clients

**FIGURE 2:**
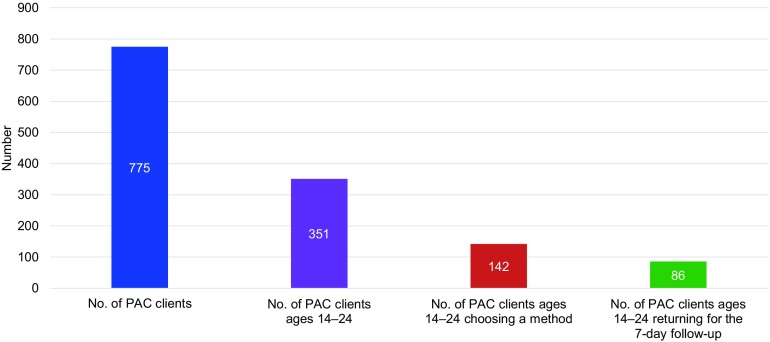
PAC Clients Choosing a Modern Contraceptive Method in All 5 Facilities, 6 Months After AFPAC Training Abbreviations: AFPAC, adolescent-friendly postabortion care; PAC, postabortion care.

Prior to the provider training, no youth PAC client had selected a contraceptive or returned to the facility for routine follow-up. Of the 775 PAC clients who sought services at any of the 5 health facilities within the 6 months after the training, 351 (45.3%) were 14 to 24 years old (Figure 2). Of these PAC clients, 142 (40.5%) chose a modern contraceptive. (It should be noted that although PAC clients aged 14 years old were included in the sample, they were not a statistically significant portion of all PAC clients. Only 2 of the facilities had any PAC clients who were 14 years old.)

**FIGURE 3:**
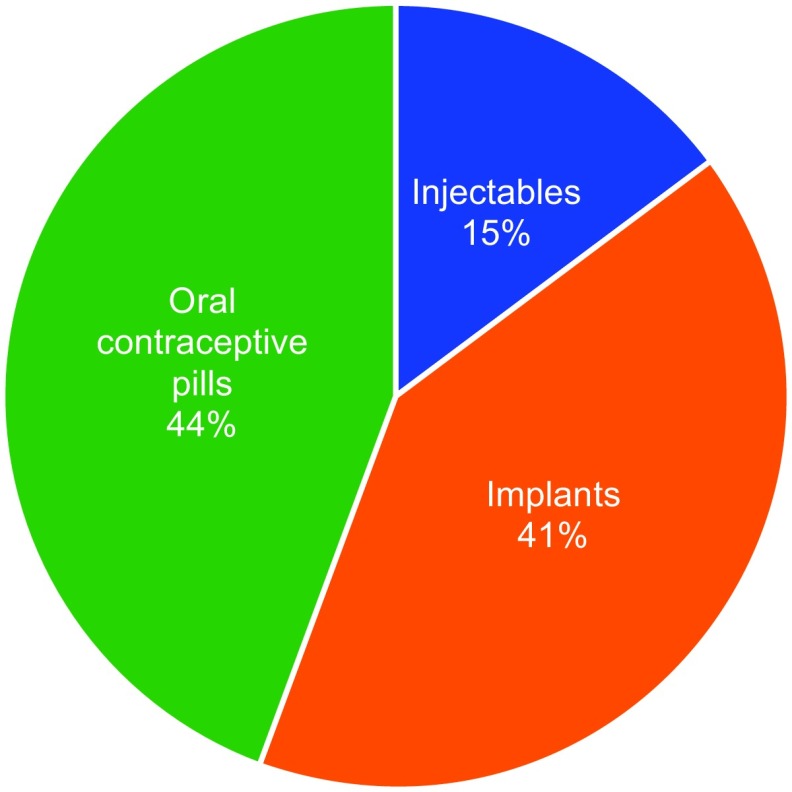
Most Commonly Chosen Contraceptive Methods by PAC clients Ages 14–24

Among the adolescent and youth PAC clients who chose a contraceptive method (n=142), the most commonly selected method was oral contraceptive pills (44%), followed by implants (41%) and injectables (15%). In addition, 86 (58.5%) of these clients returned for the 7-day follow-up (Figure 3). Providers stated that they provided information on all contraceptive methods.

## LESSONS LEARNED AND RECOMMENDATIONS

### Invest in Further Capacity Building and On-Site Support for Scale-Up

DSMI/FP adopted the AFPAC Training Module in national policies, norms, and protocols but has not been able to train other providers due to capacity limitations and the unavailability of continued support from the E2A and Agir/PF projects. Sustainability and scale-up by DSMI/PF will be contingent upon further capacity building and on-site support for transfer of learning. Continued support and documentation at the 5 E2A-supported health facilities will be necessary to assess feasibility and scalability of AFPAC to inform systematic scale-up.

### Explore Barriers to Youth Uptake of LARC

Evidence shows that sexually active adolescents and youth, regardless of marital status and parity, will use modern contraception when provided access to a wide range of contraceptive options, including LARCs. Additionally, when LARCs are chosen, continuation rates are higher than for short-acting methods and they are not subject to user error like condoms and pills.^12^ Investments should be made to explore any underlying barriers to voluntary use of LARCs among adolescents and youth while ensuring a wide range of contraceptive method choices. The low cost of oral contraceptive pills is a major contributing factor to their choice among youth because they are the least expensive method next to condoms.

### Follow Up With Supportive Supervision and Technical Support

Training alone is not sufficient to change provider attitudes and behaviors. Such shifts also require time, supportive supervision, and continued technical support. Although Agir/PF disseminated the updated policies, norms, and protocols and provided orientation on application of the youth-friendly PAC guidelines at facilities in Sokodé, Kara, and Lomé, there was no further support for geographic scale-up to other regions due to resource limitations at DSMI/PF. To enhance sustainability and potential for scale-up, DMSI/FP requires more technical support to implement the updated policies and guidelines.

### Increase Accessibility of PAC Through Linkages With Other Services

PAC can be linked with other services, such as SGBV, HIV counseling and testing, and treatment of sexually transmitted infections, as entry points for adolescent clients accessing voluntary family planning information and services. This approach will help to avoid missed opportunities and to prevent future unintended pregnancies. These services should be free or affordable.

## CONCLUSIONS

During the baseline assessment prior to the AFPAC training, it became clear that providers lacked both the willingness and the skills to offer AFPAC to their young clients, although these providers had received comprehensive training on provision of quality PAC. Use of the AFPAC Training Module improved provider attitudes, reduced bias against contraceptive use among young clients, and, for the first time, enabled providers to address SGBV. The results of this intervention revealed a need for AYRH services beyond PAC. Services tailored to meet the diverse reproductive health needs of adolescents and youth should be developed, building the competencies of providers in AYRH and ensuring the availability and use of job aids, policies and guidelines, and adequate equipment and supplies. Additionally, services need to be organized for privacy and accessibility, and they should be made free or affordable.

The training module can be used in other countries to improve PAC for adolescents and youth. However, it will be necessary to assess “youth friendliness” in provision of PAC, identify barriers to AFPAC and voluntary family planning, and design interventions that might include AFPAC training in the particular context of each country. If evidence is generated to inform design of well-planned interventions, implementation is closely documented, and learning is applied for adaptation and systematic scale-up, then AFPAC has the potential to increase voluntary contraceptive choice and uptake among adolescents and youth both regionally in West Africa, where the need is highest, and around the world.

## Notes

**Peer Reviewed**

**Funding:** The Evidence to Action (E2A) Project, under Award Number AID-OAA-A-11-00024, gratefully acknowledges the generous support of the U.S. Agency for International Development for the development of the Training Resource Package for Family Planning (TRP), dissemination of the TRP, and adaptation of the TRP in Tanzania and Uganda.
